# 

**Published:** 1913-03

**Authors:** 


### THE BRISTOL
### Medico-Chirurgical Journal.

605
400
19

A JOURNAL OF THE MEDICAL SCIENCES FOR THE
WEST OF ENGLAND AND SOUTH WALES

*PUBLISHED UNDER THE AUSPICES OF*
*THE BRISTOL MEDICO-CHIRURGICAL SOCIETY.*

EDITOR:
P. WATSON-WILLIAMS, M.D.
WITH WHOM ARE ASSOCIATED
J. MICHELL CLARKE, M.A., M.D., LL.D., J. LACY FIRTH, M.S., M.D.,
AND JAMES SWAIN, M.S., M.D.
ASSISTANT-EDITOR:
J. A. NIXON, B.A., M.B.
EDITORIAL SECRETARY:
J. M. FORTESCUE-BRICKDALE, M.A., M.D.

"Scire est nescire, nisi id me

Scire alius sciret."

LIBRARY OF CONGRESS

515646

JAN 7 1914

SMITHSONIAN DEPOSIT.

*VOL. XXXI.*

BRISTOL: J. W. ARROWSMITH LTD.
LONDON: J. & A. CHURCHILL, 7 GREAT MARLBOROUGH STREET.
1913.

R31
B8

J.W. Arrowsmith Ltd.

Publishers Bristol

### CONTENTS OF VOLUME XXXI.

*Page*

Robert Shingleton Smith, M.D., B.Sc., F.R.C.P. Editor, 1892–1912 .. 1

A Case of Acute Septicæmia due to the B. Pyocyaneus. By J. Michell Clarke, M.A., M.D., LL.D., F.R.C.P. .. .. 4

Aleucocythæmic Leukæmia. By Rupert Waterhouse, M.D. Lond., M.R.C.P. .. .. .. .. .. .. .. .. 10

Notes on a Case of Alternating Oxaluria and Phosphaturia. By F. H. Edgeworth, M.D. Cantab., D.Sc. Lond. .. .. .. 18

A Case of Alternating Oxaluria and Phosphaturia. By C. W. J. Brasher, M.D., Ch.B. Bristol .. .. .. .. .. 21

The Intravenous Administration of Neo-Salvarsan in Syphilis. By J. A. Nixon, M.B. Cantab., F.R.C.P. .. .. .. .. 24

An Example of the Stokes-Adams Syndrome. By Carey Coombs, M.D. Lond., M.R.C.P. .. .. .. .. .. .. 30

The End-Results of Cases Operated on for Gallstones. By A. Rendle Short, M.D., B.S., B.Sc. Lond., F.R.C.S. .. 34

The Treatment of Chronic Constipation: a Discussion at the Bristol Medico-Chirurgical Society, February 12th, 1913. Introduced by F. H. Edgeworth, M.D. Cantab., D.Sc. Lond. .. .. 40

On the Course of Pernicious Anæmia in Older Persons. By J. Michell Clarke, M.A., M.D., LL.D., F.R.C.P. .. .. 97

Methods of Diagnosis in Gastric Cancer. By J. M. Fortescue-Brickdale, M.A., M.D. Oxon. .. .. .. .. .. 108

Paroxysmal Œdema of the Lungs. By C. E. S. Flemming, M.R.C.S. Eng., L.R.C.P. Lond. .. .. .. .. .. 121

Notes on Two Cases of Urinary Calculus. By C. A. Moore, M.S. Lond., Ch.M. Bristol, F.R.C.S. .. .. .. .. 126

CONTENTS.

Page

Urinary Products of Intestinal Intoxication. By G. HELY-HUTCHINSON ALMOND, M.A., B.M., B.Ch. Oxon. .. .. .. .. 130

Some Recent Cases of Epidemic Cerebro-Spinal Meningitis. By J. R. CHARLES, M.A., M.D. Cantab., F.R.C.P. .. .. .. 142

On the Occurrence of Serous Pleurisy due to Pneumococci and Tubercle Bacilli. By F. H. EDGEWORTH, M.D. Cantab., D. Sc. Lond. .. .. .. .. .. .. .. .. 146

What is an Accident ? By CHARLES CORFIELD, of the Middle Temple, Barrister-at-Law, M.R.C.S., L.R.C.P. .. .. .. .. 148

The Tuberculosis Officer and his Work. By LATIMER J. SHORT, M.D., B.S. Lond., M.D. Bristol .. .. .. .. .. 153

The Third South Midland Field Ambulance. By Lieut.-Colonel A. W. PRICHARD, V.D. .. .. .. .. .. .. 159

Medicine and its Practitioners during the Earlier Years of the History of Bath. By JAMES WIGMORE, M.D. .. .. .. .. 193

Hypernephroma or Mesothelioma of the Kidney. By JAMES SWAIN, M.S., M.D. Lond., F.R.C.S... .. .. .. .. .. 213

On Nephropexy. By J. LACY FIRTH, M.D., M.S. Lond., F.R.C.S... 220

Mixed Infections. By I. WALKER HALL, M.D. Vict. .. .. 227

A Case of Cervical Rib. By RUPERT WATERHOUSE, M.D., M.R.C.P. 232

Three Cases of Ataxia. By J. M. FORTESCUE-BRICKDALE, M.A., M.D., B.Ch. Oxon. .. .. .. .. .. .. .. 235

Intermittent Swelling of the Parotid Glands. By C. W. J. BRASHER, M.D., Ch.B. Bristol .. .. .. .. .. .. .. 238

The Ethical Aspects of Physical Research. By the REV. DE LACY O'LEARY, D.D. .. .. .. .. .. .. .. 242

On Hemorrhagic Diseases of the New-born, with Report of a Case due to Duodenal Ulcer. By W. J. H. PINNIGER, M.D., B.S. Lond., M.R.C.S., L.R.C.P. .. .. .. .. .. .. .. 248

Specialism and the Medical Curriculum, mainly in Reference to Otology, Rhinology and Laryngology. By P. WATSON-WILLIAMS, M.D. Lond. .. .. .. .. .. .. .. .. 289

CONTENTS.

*Page*

Protective Ferments. By I. WALKER HALL, M.D. Vict. .. .. 304

On the Cerebral Symptoms of Lobar Pneumonia in Children. By F. H. EDGEWORTH, M.D. Cantab., D.Sc. Lond. .. .. .. 308

Medical Observations in the Clifton Zoological Gardens, with Notes on the birth of two Russian Bear Cubs in Captivity. By A. J. HARRISON, M.B. Lond. .. .. .. .. .. .. 318

Splenectomy for Splenomegaly. By J. A. NIXON, M.B. Cantab., F.R.C.P. .. .. .. .. .. .. .. .. 325

Splenectomy in Banti's Disease. By E. W. HEY GROVES, M.S.Lond., F.R.C.S. .. .. .. .. .. .. .. .. 331

Pericolitis. By T. CARWARDINE, M.S., F.R.C.S. .. .. .. 333

An Apparatus for the Intratracheal Administration of Ether. By F. E. SHIPWAY, M.A., M.D. Cantab. .. .. .. .. 341

A Combined Manometer and Safety Valve for Intratracheal Anæsthesia. By STUART V. STOCK, M.B., B.S., F.R.C.S. and J. D. FRY, B.Sc. .. .. .. .. .. .. .. 344

A Simple Apparatus for Intratracheal Anæsthesia. By E. W. HEY GROVES, M.S. Lond., F.R.C.S. .. .. .. .. .. 347

PROGRESS OF THE MEDICAL SCIENCES .. 48, 165, 256, 350

REVIEWS OF BOOKS .. .. .. .. 56, 172, 268, 365

EDITORIAL NOTE .. .. .. .. .. .. .. 371

NOTES ON PREPARATIONS FOR THE SICK .. .. 89, 180, 282

THERAPEUTIC NOTES .. .. .. .. .. .. 279, 373

THE LIBRARY OF THE BRISTOL MEDICO-CHIRURGICAL SOCIETY .. .. .. 91, 183, 285, 375

MEETINGS OF SOCIETIES .. .. .. .. .. 93, 186, 380

OBITUARY .. .. .. .. .. .. .. 94, 189, 287

LOCAL MEDICAL NOTES .. .. .. .. 95, 192, 287, 383

### The Library of the Bristol Medico-Chirurgical Society.

*The following donations have been received since the publication of the List in December.*

*February 28th*, 1913.

| | |
|---|---|
| J. Paul Bush, C.M.G. (1) .. .. .. .. | 2 volumes. |
| C. Elliott, M.D. .. .. .. .. .. | 4 ,, |
| The Editor of *The Medical Officer* (2) .. .. | 2 ,, |
| The Surgeon-General, United States Army (3) .. | 1 ,, |
| J. Taylor (4) .. .. .. .. .. | 3 ,, |

A framed picture has been presented by Mr. James Taylor.

### EIGHTY-SEVENTH LIST OF BOOKS.

The titles of books mentioned in previous lists are not repeated.

The figures in brackets refer to the figures after the names of the donors, and show by whom the volumes were presented. The books to which no figures are attached have either been bought from the Library Fund or received through the *Journal*.

**Ach, A.** .. .. .. *Beiträge zur Oesophagus-Chirurgie* .. .. .. 1913
**Allen, R. W.** .. .. *Vaccine Therapy* .. .. .. .. 4th Ed. 1912
**,, ,,** .. .. *Bacterial Diseases of Respiration* .. .. .. 1913
**Archibald, A. Balfour and R. G.** *Second Review of Recent Advances in Tropical Medicine* .. .. .. .. .. (4) 1911
**Balfour and R. G. Archibald, A.** *Second Review of Recent Advances in Tropical Medicine* .. .. .. .. .. (4) 1911
**Beatson, Sir G. T.**.. *Modern Wound Treatment* .. .. .. .. 1913
**Besche, A. de** .. *Bakteriologiscke Studier over Barnetuberkulose* 1912
**Bidwell, L. A.** .. *Minor Surgery* .. .. .. .. .. 2nd Ed. [1912]
**Bolduan, C. F.** .. *Immune Sera* .. .. .. .. .. 4th Ed. 1911
**Borradaile, L. A.** *Elementary Zoology* .. .. .. .. .. .. [1912]
**Cammidge, P. J.** .. *Glycosuria and Allied Conditions* .. .. .. 1913
**Carling, W. Turner and E. R.** *Treatment after Operation* .. .. [1912]
*Catalogue (Index-) of the Surgeon-General's Office, United States Army* (3) 2nd Series, Vol. XVII 1912
**Chaplin, A.** .. .. *Illness and Death of Napoleon Bonaparte* .. 1913
**Craig, C. F.** .. .. *The Parasitic Amœbæ of Man* .. .. .. 1911
**Crookshank, F. G.** *Flatulence and Shock* .. .. .. .. .. 1912
**Cushing, H.** .. .. *The Pituitary Body and its Disorders*.. .. [1912]
**Daniels, C. W.** .. *Tropical Medicine and Hygiene.* Part III 1912
**Elliot, R. H.** .. *Sclero-corneal Trephining in Glaucoma* .. .. [N.D.]
**Evans, W.** .. .. *Diseases of the Skin* .. .. .. .. .. [1912]
**Flexner, A.** .. .. *Medical Education in Europe* .. .. (1) 1912
**Fuchs, E.** .. .. *Text-Book of Ophthalmology* (Trans. from 12th German Edition by A. Duane) 4th Ed. 1911
**Goodall, G. L. Gulland and A.** *The Blood* .. .. .. .. .. 1912

**Gulland and A. Goodall, G. L.** *The Blood* .. .. .. .. .. 1912
**Herman, G. E.** .. *Diseases of Women.* Revised (4th) Edition by the Author, assisted by R. D. Maxwell.. 1913
**Hurry, J. B.** .. *Vicious Circles in Disease* .. .. 2nd Ed. 1913
**Laird, J.** .. .. *Notes on the Treatment of Tuberculosis* .. .. 1912
**Leftwich, R. W.** .. *Tabular Diagnosis* .. .. .. .. .. .. .. 1913
**Mackie, F. P.** .. *Sleeping Sickness* .. .. .. .. .. .. .. 1912
**Macleod, J. J. R.** .. *Diabetes : its Pathological Physiology* .. .. 1913
**Marshall, C. D.** .. *Diseases of the Eyes* .. .. .. .. .. .. [1912]
**Moon, R. O.** .. *Prognosis and Treatment of Diseases of the Heart* .. .. .. .. .. .. .. .. .. .. 1912
**Moore, N.** .. .. *The History of the Study of Medicine in the British Isles* .. .. .. .. .. .. .. .. .. 1908
**Moullin, C. M.** .. *The Biology of Tumours* .. .. .. .. .. 1913
**Nisbet, J. F.** .. *The Insanity of Genius* .. .. .. 6th Ed. 1912
**Simon, C. E.** .. *Clinical Diagnosis* .. .. .. .. .. 7th Ed. 1911
**Stephenson, S.** .. *Eye-Strain in Everyday Practice* .. .. .. .. 1913
**Turner and E. R. Carling, W.** *Treatment after Operation*.. .. .. [1912]
**Whipham, T. R. C.** *Medical Diseases of Children* .. .. .. [1912]

TRANSACTIONS, REPORTS, JOURNALS, &c.

American Journal of Obstetrics, The .. .. .. .. .. Vol. LXVI 1912

American Journal of the Medical Sciences, The .. Vol. CXLIV 1912

American Laryngological Association, Transactions of the .. .. 1912

American Ophthalmological Society, Transactions of the Vol. XIII, Part I 1912

American Surgical Association, Transactions of the .. Vol. XXX 1912

Archiv für klinische Chirurgie .. .. .. .. .. Bd. XCVIII 1912

Bristol Medico-Chirurgical Journal, The .. .. .. Vol. XXX 1912

British Journal of Children's Diseases, The .. .. .. Vol. IX 1912

British Medical Journal, The .. .. .. .. .. .. .. Vol. II for 1912

Congrès de la Société internationale de Chirurgie, 3me .. .. (1) 1911

Deutsche Zeitschrift für Chirurgie .. .. .. .. .. Bd. CXVIII 1912

Edinburgh Medical Journal .. .. .. .. .. .. .. ..N.S., Vol. IX 1912

Gazette hebdomadaire des Sciences médicales de Bordeaux Tom. XXXIII 1912

Glasgow Medical Journal, The .. .. .. .. Vol. LXXVIII 1912

Hazell's Annual .. .. .. .. .. .. .. .. .. .. .. .. .. 1913

Hospital, The .. .. .. .. .. .. .. .. .. .. .. Vol. LII 1912

Hospitals—

  Guy's Hospital Reports .. .. .. .. .. .. .. .. Vol. LXVI 1912

Journal of Mental Science, The .. .. .. .. .. .. Vol. LVIII 1912

Journal of Vaccine Therapy, The .. .. .. .. .. .. .. Vol. I 1912

Lancet, The .. .. .. .. .. .. .. .. .. .. .. .. Vol. II for 1912

Medical Directory, The .. .. .. .. .. .. .. .. .. .. .. .. 1913

Medical Officer, The .. .. .. .. .. .. (2) Vols. VII, VIII 1912

Medical Press and Circular, The .. .. .. .. .. [Vol. CXLV] 1912

Medical Review, The .. .. .. .. .. .. .. .. .. .. Vol. XV 1912

| | | |
|---|---|---|
| Nordiskt medicinskt Arkiv—Inre medicin .. .. .. | Bd. XLIV | 1911 |
| Ophthalmological Transactions .. .. .. .. .. .. | Vol. XXXII | 1912 |
| Pellagra Commission of the State of Illinois, Report of the .. .. | | 1912 |
| Practitioner, The .. .. .. .. .. .. .. .. .. .. | [Vol. LXXXIX] | 1912 |
| Prescriber, The .. .. .. .. .. .. .. .. .. .. | Vol. VI | 1912 |
| Progressive Medicine .. .. .. .. .. .. .. .. | Vols. III, IV | 1912 |
| Quarterly Journal of Medicine, The .. .. .. .. .. | Vol. V | 1911–12 |
| Revue hebdomadaire de Laryngologie .. | Tom. XXXIII, Vol. II | 1912 |
| Royal Society of Medicine, Proceedings of the .. .. | Vol. V | 1912 |
| St. Petersburger medizinische Wochenschrift .. .. .. .. .. .. | | 1911 |
| Surgery, Gynecology and Obstetrics .. .. .. .. .. .. | Vol. XV | 1912 |
| Verhandlungen der deutschen Gesellschaft für Urologie .. .. .. | | 1911 |
| Wellcome Research Laboratories, Fourth Report of .. .. .. | (4) | 1911 |
| West London Medical Journal .. .. .. .. .. .. | Vol. XVII | 1912 |
| Whitaker's Almanac .. .. .. .. .. .. .. .. .. .. .. .. .. .. | | 1913 |
| Zeitschrift für Urologie .. .. .. .. .. .. .. .. .. .. .. .. | Bd. VI | 1912 |
| Zentralblatt für Chirurgie .. .. .. .. .. .. .. .. | Bd. II for | 1912 |
| Zentralblatt für Gynäkologie .. .. .. .. .. .. | Bd. II for | 1912 |
| Zentralblatt für innere medizin .. .. .. .. .. .. | Bd. II for | 1912 |

### MEETINGS OF SOCIETIES.

### Bristol Medico-Chirurgical Society.

### Local Medical Notes.

The following are recent examination results :—

UNIVERSITY OF BRISTOL.—*M.B., Ch.B.:* Kathleen M. Cole, B. C. Eskell, A. G. Hieber. *D.P.H.:* W. Pomeroy, W. Bainbridge, S. N. Tiwary; *Part I,* P. Moxey; *Part II,* A. H. Finch.

UNIVERSITY OF LONDON.—*M.B., B.S. Group II:* E. W. Wade, F. W. Clemens.

CONJOINT EXAMINING BOARD OF ENGLAND. — *Final Examination — Medicine:* C. Kingston, G. S. Applegate.* *Midwifery:* H. B. Logan, L. Page.* *Surgery:* L. Page.*

* Denotes qualification.

**University of Bristol.**—Mr. George A. Wills and Mr. Henry H. Wills have jointly given £150,000 towards the erection of a new building fronting Queen's Road, on the site of the old Blind Asylum. It is understood that £20,000 of this will be reserved for endowment of the building. Mr. W. Melville Wills has also given £20,000 in augmentation of the General Endowment Fund of the University. The gifts are made by the three brothers in memory of their father, the late H. Overton Wills, who was the first Chancellor. The main features of the structure will be a magnificent tower nearly 200 feet high. In the new buildings will be housed the Arts Library and the Medical Library, together with offices for the administrative staff and a large Assembly Hall. It is hoped to commence the work in the spring of next year and to complete the structure in 1917.

**An Historical Medical Exhibition in London.**—In connection with the International Medical Congress to be held this summer in London, an exhibition of rare and curious objects relating to medicine, chemistry, pharmacy and the allied sciences is being organised by Mr. Henry S. Wellcome.

Among other interesting sections is one including the medical deities of savage, barbaric and other primitive peoples. Through the kindness of friends, specimens of these have been forwarded from all parts of the globe, but there are still many gaps to be filled, and those who possess such objects and would be willing to loan them should communicate with the Secretary of the Exhibition, whose address is given below.

Amulets, talismans and charms connected with the art of healing will also form another prominent feature, and any loans of this description would be welcomed.

In the section of surgery an endeavour will be made to trace the evolution and development of the chief instruments in use at the present day, and it is desired to accumulate specimens of instruments used in every part of the world by both savage and civilised peoples.

A complete illustrated syllabus will be forwarded to anyone interested on application to the Secretary, 54A Wigmore Street, London, W.

### APPOINTMENTS.

**Bristol Royal Infirmary.**—The following appointments have been made:—J. Paul Bush, C.M.G., M.Ch., to be Honorary Consulting Surgeon. E. H. E. Stack, M.B., B.C. Cantab., F.R.C.S., to be Honorary Surgeon. A. Rendle Short, M.D., B.Sc. Lond., F.R.C.S., to be Honorary Assistant Surgeon. G. I. Strachan, M.B., Ch.B. Glasgow, to be House Physician.

### Bristol Medico-Chirurgical Society.

### President.

WALTER C. SWAYNE, M.D., B.S. Lond. and Bristol.

### President-Elect.

P. WATSON-WILLIAMS, M.D. Lond.

### Committee.

*Ex-Officio*
- C. A. MORTON, F.R.C.S. (Retiring President).
- P. WATSON-WILLIAMS, M.D. Lond. (Editor of *Journal*).
- C. K. RUDGE, M.R.C.S. (Hon. Librarian).
- J. M. FORTESCUE-BRICKDALE, M.A., M.D. Oxon. (Editorial Secretary).

*Elected.*

R. SHINGLETON SMITH, M.D. Lond. } Ex-Presidents.
J. MICHELL CLARKE, M.A., M.D. Cantab. } Ex-Presidents.
G. PARKER, M.A., M.D. Cantab.
I. WALKER HALL, M.D. Vict.
F. LACE, F.R.C.S.
J. LACY FIRTH, M.D., M.S. Lond.
J. O. SYMES, M.D. Lond.
L. E. V. EVERY-CLAYTON, M.D., B.S Lond.

### Honorary Secretary.

A. J. WRIGHT, M.B., B.S. Lond.

Medical Practitioners wishing to join the Society are requested to communicate with the Hon. Secretary, Dr. A. J. WRIGHT, 60 St. Paul's Road, Clifton. The Annual Subscription is 21/-.

---

*Extracts from Journal By-laws.*

4.—The *Journal* shall be delivered free to Subscribers and also to Members of the Society whose subscriptions are not in arrear.

6.—The Annual Subscription to the *Journal* for those who pay before the 1st of July shall be 5/-, post free, or 6/- if paid after that date.

---

Communications intended for insertion in the *Journal* should be sent to the Editor, Dr. WATSON-WILLIAMS, 2 Rodney Place, Clifton, Bristol.

Books for Review and Exchange Journals should be sent to the Assistant-Editor, *Bristol Medico-Chirurgical Journal*, Medical Library, The University, Bristol.

Communications referring to the delivery of the *Journal* should be sent to the Editorial Secretary, Dr. FORTESCUE-BRICKDALE, 52 Pembroke Road, Clifton, Bristol, to whom Subscriptions are to be paid *in advance.*

Cases for Binding Vols. I—XXX are now ready, and may be obtained, price 7*d.* each (postage 2*d.* extra), upon application to J. W. ARROWSMITH LTD., Quay Street, Bristol; or the *Journal* will be bound, including Case, for 1/3 per volume.

### Bristol Medico-Chirurgical Society.

### PAST PRESIDENTS.

1874—75. FREDERICK BRITTAN, M.D., B.A. Dub.

1875—76. ROBERT WILLIAM COE.

1876—77. SAMUEL MARTYN, M.D. Ed.

1877—78. AUGUSTIN PRICHARD, M.D. Berlin.

1878—79. HENRY EDWARD FRIPP, M.D. Würzburg.

1879—80. WILLIAM MICHELL CLARKE.

1880—81. JOSEPH GRIFFITHS SWAYNE, M.D. Lond.

1881—82. EDWARD LONG FOX, M.D. Oxon.

1882—83. JAMES GEORGE DAVEY, M.D. St. And.

1883—84. WILLIAM JOHNSTONE FYFFE, M.D., B.A. Dub.

1884—85. GEORGE FORSTER BURDER, M.D. Aberd.

1885—86. WILLIAM HENRY SPENCER, M.D., M.A. Cantab.

1886—87. FRANCIS POOLE LANSDOWN.

1887—88. LEMUEL MATTHEWS GRIFFITHS.

1888—89. ROBERT SHINGLETON SMITH, M.D., B.Sc. Lond.

1889—90. NELSON CONGREVE DOBSON.

1890—91. SAMUEL HENRY SWAYNE.

1891—92. FRANCIS RICHARDSON CROSS, M.B. Lond.

1892—93. EDWARD MARKHAM SKERRITT, M.D., B.S., B.A. Lond.

1893—94. JAMES GREIG SMITH, M.B., C.M., M.A. Aberd., F.R.S.E.

1894—95. ALFRED JAMES HARRISON, M.B. Lond.

1895—96. ARTHUR WILLIAM PRICHARD.

1896—97. ALFRED EDWARD AUST LAWRENCE, M.D., C.M. Aberd.

1897—98. JOHN EDWARD SHAW, M.B., C.M. Ed.

1898—99. ROBERT ROXBURGH, M.B. Ed.

1899—00. WILLIAM HENRY HARSANT.

1900—01. DAVID SAMUEL DAVIES, M.D. Lond.

1901—02. BARCLAY JOSIAH BARON, M.B., C.M. Ed.

1902—03. GEORGE MUNRO SMITH.

1903—04. JAMES PAUL BUSH, C.M.G.

1904—05. REGINALD EAGER, M.D. Lond.

1905—06. JOHN DACRE.

1906—07. JAMES TAYLOR.

1907—08. HENRY WALDO, M.D., C.M. Aberd.

1908—09. JOHN MICHELL CLARKE, M.D. Cantab.

1909—10. JAMES SWAIN, M.D., M.S. Lond.

1910—11. BERTRAM MITFORD HERON ROGERS, B.A., M.D., B.Ch. Oxon.

1911—12. CHARLES A. MORTON.

1913.

---

### LIST OF SUBSCRIBERS

TO THE

### Bristol Medico-Chirurgical Journal.

---

### HONORARY MEMBERS OF THE SOCIETY.

| | |
|---|---|
| Owen, Sir Isambard, D.C.L., M.D. | Hereford House, Clifton. |
| Dobson, N. C., M.Ch., F.R.C.S. ... | 16 College Road, Clifton. |
| Griffiths, L. M., M.R.C.S. ... ... | 11 Pembroke Road, Clifton. |
| Smith, R. Shingleton, M.D., B.Sc., F.R.C.P. ... ... ... ... ... ... | Deepholm, Clifton Park. |

---

### SUBSCRIBERS.

*Those marked * are Members of the Society.*

| | |
|---|---|
| Ackland, J. McKno ... ... ... ... ... ... ... | 1 Barnfield Crescent, Exeter. |
| *Ackland, W. R. ... ... ... ... ... ... ... ... | 5 Rodney Place, Clifton. |
| Adye, W. J. A. ... ... ... ... ... ... ... ... .. | Church House, Bradford, Wilts. |
| Aldous, George F. ... ... ... ... ... ... ... | Charlton House, Mannamead, Plymouth |
| *Alexander, D.A., M.B., Ch.B. Vict.... ... ... | 30 Berkeley Square, Clifton. |
| *Almond, G. H. H., M.A., M.B., B.Ch. Oxon. | Lynvale, St. Mark's Place, Bath. |
| *Aubrey, T., M.B. Lond. ... ... ... ... ... | Bitton, near Bristol. |
| Aveline, H. T. S. ... ... ... ... ... ... ... ... | Somerset Asylum, Taunton. |

| | |
|---|---|
| *Baker, Lily A., M.B., B.Ch., B.A.O., R.U.I.... | 3 Whiteladies Road, Clifton. |
| *Ballance, H. Stanley M.D. Lond. ... ... | 4 Royal Terrace, Weston-super-Mare. |
| Barber, M. C. ... ... ... ... ... ... ... ... | Staple Hill, Bristol. |
| Baretti, T. G. L'Enard ... ... ... ... ... .. | 49 Royal York Crescent, Clifton. |
| *Barker, G. H., M.D., B.Sc. Lond. ... ... ... | 124 Redland Road, Bristol |
| *Baron, Barclay J., M.B., C.M. Ed.... ... ... | 16 Whiteladies Road, Clifton. |
| Basset, Walter ... ... ... ... ... ... ... | 24 Stow Hill, Newport, Mon. |
| Batten, W. Smith... ... ... ... ... ... ... | West Hill, Calne. |

### LIST OF SUBSCRIBERS.

Bayer Co., The ... ... ... ... ... ... ... 19 St. Dunstan's Hill, London, E.C.
*Benson, J. R. ... ... ... ... ... ... ... ... 11 The Circus, Bath.
*Bergin, F. G. ... ... ... ... ... ... ... ... 12 West Park, Clifton.
Bernard, C. ... ... ... ... ... ... ... ... 2 Spencer Terrace Fishponds.
*Berry, F. C., M.D., B.Ch., B.A. Dub. ... ... Burnham, Somerset.
Berry, James, M.B., B.S. Lond. ... ... ... 21 Wimpole Street, London, W.
Biamonti, Sebastiano ... ... ... ... ... ... 27 Via Balbi, Genoa, Italy.
*Blacker, A. E.... ... ... ... ... ... ... ... 20 Victoria Square, Clifton.
Blackett, J. F., M.D., B.S. Lond. ... ... ... Audley, Newcastle, Staffs.
*Blacklry, F. J., M.D. Dub. ... ... ... ... 2 Knowle Road, Bristol.
*Blagg, Arthur F., M.D. Durh.... ... ... ... 28 Caledonia Place, Clifton.
Bodman, Hervey, M.D. Lond. ... ... ... ... 9 Whiteladies Road, Clifton.
*Boucher, F. T. ... ... ... ... ... ... ... ... 31 Downleaze, Stoke Bishop.
*Bowker, G. E., M.D. Edin.... ... ... ... ... 11 North Parade, Bath.
Boyce, James G. ... ... ... ... ... ... ... The Old Hall, Biddenden, Kent.
Boyd, Geo. S. J. ... ... ... ... ... ... ... 19 St. George's Square, Stamford, Lincs.
*Brasher, C. W. J., M.B., Ch.B. Bristol ... ... 128 Cotham Brow, Bristol.
*Brew, R. H. ... ... ... ... ... ... ... ... Chew Magna.
*Bristowe, H. C., M.D. Lond. ... ... ... ... Wrington, Somerset.
*Brown, William, M.D., C.M. Glasg. ... ... Park View, Fishponds, Bristol.
*Bullen, F. St. John ... ... ... ... ... ... 3 Richmond Park Road, Clifton.
*Burdon-Cooper, J., M.D., B.Sc. Durh. ... ... 12 The Circus, Bath.
Burroughs, E. F. H. ... ... ... ... ... ... 27 Burlington Road, Redland, Bristol.
*Bush, J. Paul, C.M.G. ... ... ... ... ... ... Vyvyan House, Clifton Park, Clifton.

*Cadman, E. S. R., M.D., C.M. Edin. ... ... 22 Trelawney Road, Cotham.
*Carling, A., M.A., M.B., B.C. Cantab. ... ... 12 Great George Street, Bristol.
*Carter, T. M., M.D. Durh. ... ... ... ... Burnley, Westbury-on-Trym.
*Carwardine, Thomas, M.B., M.S. Lond. ... 16 Victoria Square, Clifton.
*Cave, Edward J., M.D. Lond. ... ... ... ... 20 Circus, Bath.
*Charles, J. R., M.A., M.D., B.C. Cantab. ... 12 Victoria Square, Clifton.
*Chitty, H., M.S. Lond. ... ... ... ... ... 46 Pembroke Road, Clifton.
Chute, Henry M. ... ... ... ... ... ... ... King William's Town, S. Africa.
*Clarke, C., M.B., B.S. Lond. ... ... ... ... Brompton Hospital, London.
*Clarke, John-Michell, M.D., M.A. Cantab. 28 Pembroke Road, Clifton.
*Clarke, R. C., M.B., Ch.B. Bristol ... ... ... Royal Infirmary, Bristol.
Cobbledick, A. S., M.D., B.S. Lond. ... ... 402 Brixton Road, London, S.W.
Connellan, Lieut. P. S., I.M.S. ... ... ... c/o Grindlay & Co., 54 Parliament Street, London.
*Cook, Henri, M.D., C.M. Aberd. ... ... ... 1 Dean Lane, Southville, Bristol.
*Coombs, Carey, M.D. Lond. ... ... ... ... 3 Pembroke Road, Clifton.
*Cooper, William Russell ... ... ... ... 12 Whiteladies Road, Clifton.
*Corfield, C. .. ... ... ... ... ... ... ... 12 Pembroke Road, Clifton.
Cossham, W. R., M.D., C.M. Aberd.... ... ... Cirencester.
Cotton, William, M.D., C.M., M.A. Ed. ... 231 Gloucester Road, Bristol.
Coulter, R. J., M.B. Dub. ... ... ... ... ... 11 Clytha Park Road, Newport, Mon.
Cridland, A. B. ... ... ... ... ... ... ... 52 Waterloo Road, Wolverhampton.
*Cross, F. Richardson, M.B. Lond.... ... ... Worcester House, Clifton.
*Crossman, F. W. ... ... ... ... ... ... ... Hambrook, Gloucestershire.
*Crouch, C. Percival, M.B. Lond. ... ... ... 1 Royal Terrace, Weston-super-Mare.

*Dacre, John ... ... ... ... ... ... ... ... 14 Eaton Crescent, Clifton.
*Davies, D. S., M.D. Lond., D.P.H. Cantab. ... 60 Oakfield Road, Clifton.

Dening, Edwin ... ... ... ... ... ... ... ... Manor House, Stow-on-the-Wold.
Devine, H., M.D., Lond. ... ... ... ... ... ... West Riding Asylum, Wakefield.
Dickie, James Steel, M.D., C.M. Aberd. ... Boscombe Park, Bournemouth.
*Dickinson, W. G., M.D., Durh. ... ... ... Wood Hill, Adelaide Terr., Portishead.
*Dixon, T. B. ... ... ... ... ... ... ... ... ... 134 Coronation Road, Bristol.
*Dowling, E. A. G. ... ... ... ... ... ... ... 5 Rodney Place, Clifton.
*Duckett, A. H., M.B. ... ... ... ... ... ... ... Elm Grove Infirmary, Brighton.
Dunbar, Eliza L. Walker, M.D. Zürich ... 9 Oakfield Road, Clifton.
Dymoke, F. ... ... ... ... ... ... ... ... 21 Cotham Road, Bristol.

*Eager, Reginald, M.D. Lond. ... ... ... ... ... Northwoods, Winterbourne.
*Edgeworth, F. H., M.D., B.C., B.A. Cantab., D.Sc. Lond. ... ... ... ... ... ... ... ... ... 4 Richmond Park Road, Clifton.
Edwards, E. S. ... ... ... ... ... ... ... ... Empingham, Stamford.
Elder, William ... ... ... ... ... ... ... ... 4 Trelawney Road, Cotham.
Elliot Bros. Ltd. ... ... ... ... ... ... ... *Australian Pharmaceutical Notes & News*, O'Connell Street, Sydney, N.S.W.
*Elliott, Christopher, M.D., B.A. Dub. ... 102 Pembroke Road, Clifton.
Elliott, F. Percy, M.B., C.M. Aberd. ... ... 113 Grove Road, Walthamstow, London. N.E.
*Every-Clayton, L. E. V., M.D. Lond. ... ... Rosslyn, Clevedon, Somerset.

*Faill, C. J. C., M.B., B.Ch. Edin. ... ... ... Tuberculosis Dispensary, Redcliffe Parade, Bristol.
*Farquhar, J., M.D. Aberd. ... ... ... ... ... ... 11 Dublin Crescent, Henleaze Road, Bristol.
*Fawn, G. F., B.D.S. Bristol ... ... ... ... 25 Whiteladies Road, Clifton.
*Fells, A., M.B., C.M. Edin. ... ... ... ... 107 Ashley Road, Bristol.
Ferguson, R. S., B.A. Durh., M.B., C.M. Edin. Elm Grove, Calne, Wilts.
*Firth, J. L., M.D., M.S. Lond. ... ... ... ... 8 Victoria Square, Clifton.
*Fisher, A. G. T. ... ... ... ... ... ... ... ... 33 Caledonia Place, Clifton.
*Flemming, A. L., M.B., Ch.B. Bristol ... ... 48 Pembroke Road, Clifton.
*Flemming C. E. S. ... ... ... ... ... ... ... Manvers House, Bradford-on-Avon.
*Fortescue-Brickdale, J. M., M.A., M.D., B.Ch. Oxon. ... ... ... ... ... ... ... ... 52 Pembroke Road, Clifton.
Foss, E. V., M.B., Ch.B. Bristol ... ... ... Clouds Hill House, St. George, Bristol.
*Fraser, Forbes ... ... ... ... ... ... ... ... 2 The Circus, Bath.
Freeman, J., M.D. Brux. ... ... ... ... ... Waterloo Villa, Queen's Road, Clifton.

Garland, E. B., M.B., C.M. Edin. ... ... ... 114*a* Hampton Road, Redland.
Gee, C. A. H., M.B., Ch.B. Bristol ... ... ... Evercreech, Somerset.
Genge, T. T. ... ... ... ... ... ... ... ... ... 21 Whiteladies Road, Bristol.
*Gerrish, D. S. ... ... ... ... ... ... ... ... ... 322 Church Road, St. George, Bristol.
*Gibbs, A. N. Godby ... ... ... ... ... ... ... 52 Whiteladies Road, Clifton.
*Goodden, H. W., M.B., Ch.B. Bristol ... ... The Royal Infirmary, Bristol.

*Gratte, C. Brooke ... ... ... ... ... ... ... 19 Gold Tops, Newport, Mon.
*Green, T. A., M.D., C.M. Edin. ... ... ... 33*a* Whiteladies Road, Clifton.
Green-Armytage, Capt. V. B., I.M.S. ... ... c/o King & Co., Bombay.
*Greer, W. J. ... ... ... ... ... ... ... ... ... 19 Gold Tops, Newport, Mon.
Griffiths, Cornelius A. ... ... ... ... ... ... 6 Windsor Place, Cardiff.
*Griffiths, J. S. ... ... ... ... ... ... ... 20 Redland Park, Bristol.
*Groves, E. W. H., M.D., M.S. Lond. ... ... 16 Richmond Hill, Clifton.

*Hailes, Clements D. G., M.D., C.M. Ed. ... 27 Alma Road, Clifton.
*Hall, E. G., M.B. Lond. ... ... ... ... ... ... 53 Hampton Road, Redland, Bristol.
*Hall, I. Walker, M.D., Ch.B.Vict. ... ... Linden Gate, Clifton Down Road, Clifton.
Handfield-Jones, Montagu, M.D. Lond. ... 35 Cavendish Square, London, W.
*Harris, H. Elwin, B.A., M.B. Cantab. ... ... 13 Lansdown Place, Clifton.
*Harsant, W. H. ... ... ... ... ... ... ... ... Tower House, Clifton.
*Harty, J. P. I., M.B., B.Ch., B.A.O., R.U.I. Bristol Royal Infirmary.
*Hawes, C. S. ... ... .. ... ... ... ... ... Bramshot, Liphook, Hants.
*Hawes, I. H., M.B., B.S. Durh. ... ... ... Wick, Glos.
Hayman, Alfred G. ... ... ... ... ... ... Hapsford House, near Frome.
*Hayman, C. A., M.D. St. And. ... ... ... ... Kingston Villa, Richmond Hill, Clifton.
Hele, T. S., M.A., M.B. ... ... ... ... ... ... Emmanuel College, Cambridge.
*Herapath, C. E. K., M.D. Lond. ... ... ... 12 Brunswick Square, Bristol.
*Herapath, C. K. C. ... ... ... ... ... ... ... Cotham Park, Bristol.
Hill, Hedley, M.D. Durh. ... ... ... ... 1 Whiteladies Road, Clifton.
*Hill, Walter J. ... ... ... ... ... ... ... ... The Brow, Clevedon.
Hospital, Bristol General (*Sec.*, W. Thwaites).

*Iles, A. E., M.B., B.S. Lond. ... ... ... ... Cotham Lawn, Cotham Park, Bristol.
*Imlay, G. A., M.B., C.M. Aberd. ... ... ... 1 Cotham Park, Bristol.
Infirmary, Bristol Royal (*Secretary*, W. E. Budgett).
Ingle, C. Durban ... ... ... ... ... ... ... ... Somerton, Somerset.
*Irwin, S., M.B., B.Ch., B.A.O., R.U.I. ... ... Olveston, Glos.

*James, C. W. W. ... ... ... ... ... ... ... ... 246 Wells Road, Knowle.
Jamison, A., M.B., B.Ch., M.A. R.U I. ... ... Ardenvohr, Woodstock Road, Belfast.
*Joll, C.A., M.B., M.S. Lond. ... ... ... ... 14 Elton Terrace, Bishopston.
*Jones, E. J. Trevor, M.D. Brux. ... ... ... Aberdare, Glamorganshire.

| | |
|---|---|
| *Jones, J. Ellington ... ... ... ... ... .. ... | 16 Richmond Park Road, Clifton. |
| Jones, H. Macnaughton, M.D., M.Ch.R.U.I. | 131 Harley Street, London, W. |
| Joseph, A. Hill, M.D. Lond. ... ... ... ... | Bexhill-on-Sea. |

| | |
|---|---|
| *Kay-Mouat, J. R., M.A. Oxon., M.B., Ch.B. Bristol ... ... ... ... ... ... ... ... ... | 3 Gloucester Row, Clifton. |
| *Kemm, F. St. J. ... ... ... ... ... ... ... ... | Worle. |
| *Kingston, S. H., M.B., Ch.B. Bristol ... ... | Royal Infirmary, Bristol. |
| *Kyle, H. G., M.B., B.Ch. Oxon. ... ... ... | 31 Westbury Road, Westbury-on-Trym. |

| | |
|---|---|
| *Lace, F. ... ... ... ... ... ... ... ... ... | 5 Gay Street, Bath. |
| *Lansdown, R. G. P., M.B., B.S. Dur. ... ... | 39 Oakfield Road, Clifton. |
| *Lavington, C. C., M.B., B.S. Dur. ... ... | 1 Burlington Road, Redland. |
| Lendon, A. A., M.D. Lond. ... ... ... ... | North Terrace, Adelaide, S. Australia. |
| Le Soudier, H. ... ... ... ... ... ... ... | 174 et 176 Boulevard St. Germain, Paris. |
| *Lees, E. Leonard, M.D., C.M. Ed. ... ... ... | 1 Chesterfield Place, Clifton. |
| Leigh, W. W. ... ... ... ... ... ... ... ... ... | Treharris, Glamorganshire. |
| *Leonard, R. C., M.D. Ed. ... ... ... ... ... | 91 Coronation Road, Bristol. |
| *Llewellyn, R. Ll. J., M.B. Lond. ... ... ... | 41 Gay Street, Bath. |
| *Lucas, J. J. S., M.D. Lond. ... ... ... ... ... | 186 Wells Road, Totterdown. |
| Lucy, Reginald H. ... ... ... ... ... ... | 9 The Crescent, Plymouth. |

| | |
|---|---|
| MacDonald, P. W., M.D., C.M. Aberd. ... ... | Herrison, Dorchester. |
| Mackinnon, Charles, M.B., C.M., M.A. Glasg. | Davaar, Cirencester. |
| Maclean, Ewen J., M.D., C.M. Ed. ... ... ... | 12 Park Place, Cardiff. |
| Maclean, J. Campbell, M.B., C.M. Ed. ... ... | Swindon House, Swindon, Wiltshire. |
| *Macleod, D., M.B., Ch.B. Ed. ... ... ... ... | 84 Redland Road, Bristol. |
| *Macphail, J. M., M.B., Ch.B. Ed. ... ... ... | Eastern Dispensary, Bath. |
| *MacWatters, J. C. .. ... ... ... ... ... ... | Almondsbury. |
| Marsh, O. E. Bulwer ... ... ... ... ... ... | Parkdale, Newport, Mon. |
| Marshall, Arthur L., M.B., B.A. Cantab. ... | 206 Upper Clapton Road, London, N.E. |
| *Martin, E. F., M.B. Ed. ... ... ... ... ... | 7 Royal Terrace, Weston-super-Mare. |
| *Martin, R. C. ... ... ... ... ... ... ... ... ... | 3 Clifton Vale, Clifton. |
| *Martyn, G. J. King, M.D., B.C., B.A. Cantab. ... ... ... ... ... ... ... ... ... | 12 Gay Street, Bath. |
| Mason, S. B. ... ... ... ... ... ... ... ... ... | 12 Park Terrace, Pontypool. |
| Miall, C. L. ... ... ... ... ... ... ... ... ... | Elm Hayes, Paulton, near Bristol. |
| *Mole, Harold F. ... ... ... ... ... ... ... | 19 Mortimer Road, Clifton, Bristol. |

*MOORE, C. A., M.B., M.S. Lond. ... ... ... ... 56 St. Paul's Road, Clifton.
*MORTON, C. A. ... ... ... ... ... ... ... ... ... 14 Vyvyan Terrace, Clifton.
*MUMFORD, W. G., M.B. Lond. ... ... ... ... 18 The Circus, Bath.
MUNDEN, CHARLES ... ... ... ... ... ... ... ... Ilminster.
*MUNRO, J. M. H., D.Sc. Lond. ... ... ... ... 12 Grosvenor Place, Bath.
*MYLES, G T. ... ... ... ... ... ... ... ... ... 7 Apsley Road, Clifton.

*NEILD, NEWMAN, M.B., B.Ch. Vict. ... ... ... 9 Richmond Hill, Clifton.
*NEWMAN, HERBERT, M.B., B.S.Dur. ... ... 150 Wells Road, Bristol.
*NEWNHAM, W. H. C., M.B., M.A. Cantab. ... 3 Lansdown Place, Victoria Square, Clifton.
*NICHOLS, F. C., M.B., Ch.B. Bristol ... .. ... 38 Whiteladies Road, Clifton.
*NIXON, J. A., B.A., M.B., B.C. Cantab. ... ... 19 Mortimer Road, Clifton.

ODELL, WILLIAM, M.D. Durh. ... ... ... ... Ferndale, Torquay.
*OGILVY, ALEXANDER, M.D., B.Ch., B.A. Dub. Leny, Clifton Park, Clifton.
OPENSHAW, E. H. ... ... ... ... ... ... ... ... Cheddar.
OPPENHEIMER, SON & CO. LTD. ... ... ... 179 Queen Victoria Street, E.C.
*ORMEROD, H. LAWRENCE, M.B., B.Ch. R.U.I. 2 Henleaze Road, Westbury Park.

PAGE, G. SHEPLEY ... ... ... ... ... ... ... ... 78 Old Market Street, Bristol.
*PARKER, GEORGE, M.D., M.A. Cantab. ... ... 14 Pembroke Road, Clifton.
PARKINSON, Lt. G. S., R.A.M.C. ... ... ... c/o Messrs. Holt & Co., Whitehall, S.W.
*PARSONS, H. F.... ... ... ... ... ... ... ... The Heath, Stoke Bishop.
PARSONS, J. H., M.B., B.S., D.Sc. Lond.... ... 54 Queen Anne Street, London, W.
PATERSON, DONALD ROSE, M.D., C.M. Ed. ... 18 Windsor Place, Cardiff.
PEAKE, G. ARTHUR ... ... ... ... ... ... ... Alma House, Cheltenham.
*PEARSE, J., M.D., C.M. Ed.... ... ... ... ... Sarnia, Trowbridge.
PHILLIPS, C. M., M.D. Brux. ... ... ... ... Ravenslea, Kensington Hill, Brislington.
*PICKERING, C. F. ... ... ... ... ... ... ... ... 13 Berkeley Square, Bristol.
*PINNIGER, W. J. H., M.D., B.S. Lond. ... ... 75 Effingham Road, Bristol.
*PINEO, E. G. D.... ... ... ... ... ... ... ... ... Richmond House, Langford, nr. Bristol.
*PORTEOUS, H. B., M.B., Ch.B. Ed. ... ... ... Newbury House, Weston-super-Mare.
POWELL, J. J., M.D. Lond. ... ... ... ... ... 2 Gloucester Road, Bishopston, Bristol.
POWELL, WILLIAM, M.B. Lond. ... ... ... ... Hill Garden, Torquay.
PRICE, D. T. M.B. Lond. ... ... ... ... ... Castle Cary, Somerset.

*PRICHARD, ARTHUR W. ... ... ... ... ... ... 6 Rodney Place, Clifton.
*PROWSE, ARTHUR B., M.D. Lond. ... ... ... 5 Lansdown Place, Clifton.
PROWSE, W. B.... ... ... ... ... ... ... ... 31 Vernon Terrace, Brighton

RANDOLPH, CHARLES ... ... ... ... ... ... St. Michael's, Milverton, Somerset.
*RATTRAY, J. M., M.D., C.M., M.A. Aberd. ... Frome.
*RAYNER, D. C. ... ... ... ... ... ... ... ... 9 Lansdown Place, Clifton, Bristol.
RENDALL, JOHN ... ... ... ... ... ... ... Forestside, Lymington.
*RICHARDSON, T. A. ... ... ... ... ... ... ... 10 Clifton Park Road.
RITCHIE, THOMAS P. ... ... ... ... ... ... 27 Oakfield Road, Clifton.
*ROBERTSON, D., M.B., Ch.B. Edin. ... ... ... 188 Wells Road, Knowle.
*ROGERS, B. M. H., M.D., B.Ch., B.A. Oxon. ... 1 Victoria Square, Clifton.
*ROLFE, R., B.A., M.B., B.C. Cantab. ... ... Park House, Avonmouth.
*ROSE, F. H. ... ... ... ... ... ... ... ... 13 Westfield Park, Clifton.
*ROSSITER, GEORGE F., M.B. Lond. ... ... ... Cairo Lodge, Weston-super-Mare.
*ROXBURGH, ROBERT, M.B. Edin. ... ... ... 1 Victoria Buildings, Weston-super-Mare.
*RUDGE, C. KING ... ... ... ... ... ... .. 145 Whiteladies Road, Bristol.
*RUTHERFORD, J. M., M.B., C.M. Edin. ... ... Brislington House, Bristol.

SCOTT, BATY- ... ... ... ... ... ... ... 28 Belluton Road, Knowle, Bristol.
SEWARD, W. J., M.B. Lond. ... ... ... ... 15 Chandos Avenue, Oakleigh Park, London, N.
SHEPPARD, Lieut. A. L., I.M.S., M.B., B.S. Dur. c/o Grindlay, Groom & Co., Bombay.
*SHORT, A. R., M.D., B.S., B.Sc. Lond. ... ... 2 Westbourne Place, Clifton.
*SHORT, L. J., M.D., B.Sc. Lond. ... ... ... Westfield, South Road, Weston-super-Mare.
SKELTON, HENRY, M.D. St. And. ... ... ... Downend, Gloucestershire.
SMITH, Capt. F. SHINGLETON, I.M.S. ... ... c/o Grindlay & Co., Bombay.
*SMITH, G. COCKBURN, M.D. Brux. ... ... ... 14 South Road, Newton Abbot.
*SMITH, G. MUNRO ... ... ... ... ... ... ... 18 Apsley Road, Clifton.
SMITH, L. SHINGLETON, B.A., M.B. Cantab.... Camden Road, Brecon.
SMITH, REV. REGINALD, M.A. Cantab. ... ... The Vicarage, Walpole St. Andrew, Norfolk.
SMITH, W. STEELE, M.B. Lond. ... ... ... 1 Wilbraham Rd., Chorlton-cum-Hardy, Manchester.
*SMITH, W. ALEXANDER, M.B., M.A. Oxon. ... 70 Pembroke Road, Clifton.
SOCIETY, CARDIFF MEDICAL (*Hon. Lib.*, Queen Street, Cardiff).

| | |
|---|---|
| Society, Manchester Medical ... ... ... | Medical Society's Library, The Owens College, Manchester. |
| *Stack, E. H. E., B.A., M.B., B.C. Cantab. ... | Arvalee, Clifton Down Road, Clifton. |
| Staple, J. D. ... ... ... ... ... ... ... ... | 153 Cheltenham Road, Bristol. |
| Statham, R. Whiteside ... ... ... ... ... | Cheddar, Somersetshire. |
| *Statham, R. S. S., M.B., Ch.B. Bristol ... ... | The Royal Infirmary, Bristol. |
| Stevens, W. H. ... ... ... ... ... ... ... | 26 Zetland Road, Bristol. |
| *Stock, W. S. V., M.B. B.S Lond. ... ... ... | Montrose House, Queen's Road, Clifton |
| Stoddart, F. Wallis ... ... ... ... ... ... | Southey House, College Green, Bristol. |
| *Swain, James, M.D., M.S. Lond. ... ... ... | 4 Victoria Square, Clifton. |
| *Swayne, W. Carless, M.D. Lond. ... ... ... | 2 Clifton Park, Clifton. |
| *Symes, J. O., M.D. Lond. ... ... ... ... ... | 71 Pembroke Road, Clifton. |
| Symonds, H. P. ... ... ... ... ... ... ... | 35 Beaumont Street, Oxford. |

| | |
|---|---|
| Tayler, H. P., M.B., B.C. Cantab. ... ... ... | The Abbey House, Bradford-on-Avon. |
| Taylor, A. L. ... ... ... .. ... ... ... ... | 22 Downfield Road, Clifton |
| *Taylor, James ... ... ... ... ... ... ... ... | 36 Alma Road, Clifton. |
| *Taylor, J. W., M.B., Ch.B. Bristol ... ... ... | 8 West Redcliff Parade, Bristol. |
| *Temple, G. H., M.B., C.M. Ed. ... ... ... | Ailanthus, Weston-super-Mare. |
| Thin, James ... ... ... ... ... ... ... ... | 54 & 55 South Bridge, Edinburgh. |
| Thomas, Sir A. Garrod, M.D., C.M. Ed. ... | Clytha Park, Newport, Mon. |
| *Thomas, J. D., M.B. Cantab. ... ... ... ... | Northwoods, Winterborne, |
| Thomas, J. Lynn, C.B. ... ... ... ... ... ... | 21 Windsor Place, Cardiff. |
| *Thomas, J. M. M. ... ... ... ... ... ... ... | 5 Colston Parade, Redcliff, Bristol. |
| *Thomas, J. Tubb, D.P.H. .. ... ... ... ... | The Halve House, Trowbridge. |
| Thomas, R. E., M.D., B.S. Lond. ... ... ... | The Mount, Newport, Mon. |
| Thomson, Eustace B., M.D., C.M. Glasg. ... | Albany Place, Plymouth. |
| *Thomson, F. G., M.A., M.D. Lond. ... ... ... | 20 Gay Street, Bath. |
| Tosswill, Leonard R., D.P.H., Cantab. ... | Devon County Council Offices, 14 Bedford Circus, Exeter. |

| | |
|---|---|
| Vachell, Herbert R., M.D., C.M. Aberd. ... | 18 Newport Road, Cardiff. |
| *Vickery, W. H. ... ... ... ... ,,, ... ... | 1 Trewartha Park, Weston-super-Mare. |

| | |
|---|---|
| *Waldo, Henry, M.D., C.M. Aberd. ... ... ... | 19 Pembroke Road, Clifton. |
| *Walker, Cyril H., M.B., B.A. Cantab. ... ... | 8 Oakfield Road, Clifton. |
| *Wallace, John ... ... ... ... ... ... ... | Lauriston, 15 Knightstone Road, Weston-super-Mare. |
| Wallace, J. T., M.B., B.Ch., B.A. R.U.I. ... | 48 Coronation Road, Bedminster. |
| *Wallace, James W. ... ... ... ... ... ... | 1 Greville Road, Southville, Bristol. |
| *Walters, C. F. ... ... ... ... ... ... ... | 5 Mortimer Road, Clifton. |
| *Waterhouse, R., M.D. Lond. ... ... .. ... | 25 Circus, Bath. |
| *Watson-Williams, P., M.D. Lond.... ... ... | 2 Rodney Place, Clifton. |
| Webber, William W. ... ... ... ... ... ... | Crewkerne. |

| | |
|---|---|
| *Whicher, A. H. ... ... ... ... ... ... ... | 115 Queen's Road, Clifton. |
| White, C. R. ... ... ... ... ... ... ... ... | Nixon Villa, Merthyr Vale, Glamorgan. |
| White, J. W. ... ... ... ... ... ... ... ... | Orchard House, Nailsea. |
| *White, Percy W. ... ... ... ... ... ... ... | College Green, Bristol. |
| Wigmore, J., M.D. R. U. I.... ... ... ... ... | 16 Queen Square, Bath. |
| Willcox, R. Lewis ... ... ... ... ... ... | Warminster. |
| Willett, George G. D. ... ... ... ... ... | Keynsham, Somerset. |
| *Williams, E. C., M.B., B.C., B.A. Cantab. ... | 159 Whiteladies Road, Clifton. |
| Williams, H. Egerton ... ... ... ... ... | The Mount, Newport, Mon. |
| Williams, S. R., M.B. Lond. ... ... ... ... | Buckfastleigh, Devon. |
| *Williams, W. Roger ... ... ... ... ... ... | Morven, Walton-by-Clevedon. |
| *Williamson, G. Scott... ... ... ... ... ... | Bristol General Hospital. |
| Willis, W. M.... ... ... ... ... ... ... ... | 7 Regent Street, Nottingham. |
| *Wills, W. K., M.B., B.C. Cantab. ... ... ... | 19 Whiteladies Road, Clifton. |
| *Windsor-Aubrey, H. W. ... ... ... ... ... | Coronation Road, Bristol. |
| *Wood, W. V. ... ... ... ... ... ... ... ... | Langford, near Bristol. |
| Woollcombe, Walter L. ... ... ... ... ... | 16 The Crescent, Plymouth. |
| *Wright, A. J., M.B., B.S. Lond. ... ... ... | 60 St. Paul's Road, Clifton. |

| | |
|---|---|
| Young, J., M.D. Glas. ... ... ... ... ... ... | 14 Chantry Road, Clifton. |

### Bristol Medico-Chirurgical Journal,

MARCH, 1913.

### EXCHANGE-LIST.

### AFRICA.

CAPE COLONY.

*Twice a month.*

*South African Medical Record.* Cape Town: Townshend, Taylor & Snashall.

### AMERICA.

ARGENTINE REPUBLIC.

*Weekly.*

*La Semaña Médica.* Buenos Ayres: 737 Callao.

CANADA

*Monthly.*

*The Canada Lancet.* Toronto: The Ontario Publishing Co. Ltd.

*The Canadian Medical Association Journal.* Toronto: Morang & Co. Ltd.

MEXICO.

*Twice a month.*

*Gaceta Médica.* Mexico: Apartado postal, 517.

PERU.

*Twice a month.*

*La Crónica Médica.* Lima: Apartado postal, No. 629.

UNITED STATES.

*Weekly.*

*Boston Medical and Surgical Journal.* Boston: 101 Tremont Street.

*Medical Record.* New York: William Wood & Co.

*The Lancet-Clinic.* Cincinnati: University Building.

*Monthly.*

*American Journal of Surgery.* New York: 92 William Street.

*American Medicine.* Burlington: 189 College Street.

*Archives of Pediatrics.* New York. E. B. Treat & Co.

*Buffalo Medical Journal.* Buffalo: 228 Summer Street.

*Bulletin of the Johns Hopkins Hospital.* Baltimore: The Johns Hopkins Press.

*California State Journal of Medicine.* San Francisco: Butler Building.

*New York State Journal of Medicine.* New York: 17 West 43rd Street.

*The American Journal of Obstetrics* New York: William Wood & Co.

*The American Journal of Ophthalmology.* St. Louis: 316 Metropolitan Building.

*The Monthly Cyclopædia of Practical Medicine.* Philadelphia: The F. A. Davis Co.

*The Post-Graduate.* New York: 2nd Avenue and 20th Street.

*The Therapeutic Gazette.* Detroit: E. G. Swift.

*Quarterly.*

*The Alienist and Neurologist.* St. Louis: 3858 West Pine Boulevard.

*Irregularly.*

*The Journal of Experimental Medicine.* New York: The Rockefeller Institute for Medical Research.

*The Journal of Medical Research.* Boston: 240 Longwood Avenue.

*Yearly.*

*Smithsonian Report.* Washington: Government Printing Office.

*Studies from the Department of Pathology of the College of Physicians and Surgeons—Columbia University,* New York: 437 West 59th Street.

*Transactions of the American Association of Obstetricians and Gynecologists.* Philadelphia: William J. Dornan.

*Transactions of the American Dermatological Association.* New York: The Grafton Press.

*Transactions of the American Laryngological Association.* New York: The McConnell Printing Co.

*Transactions of the American Ophthalmological Society.* Hartford: The Case, Lockwood & Brainard Co.

*Transactions of the American Pediatric Society.* New York: E. B. Treat & Co.

*Transactions of the American Surgical Association.* Philadelphia: William J. Dornan.

*Transactions of the Association of American Physicians.* Philadelphia: William J. Dornan.

*Transactions of the College of Physicians of Philadelphia.* Philadelphia: William J. Dornan.

### ASIA

CHINA.

*Quarterly*

*The China Medical Missionary Journal.* Shanghai: 18 Peking Road.

INDIA.

*Monthly.*

*The Antiseptic.* Madras: U. Rama Rau.

*The Indian Medical Gazette.* Calcutta: Thacker, Spink & Co.

*Practical Medicine* Delhi: Egerton Street.

JAPAN.

*Monthly.*

*The Sei-I-Kwai Medical Journal.* Tokyo: Charity Hospital Medical College.

PHILIPPINE ISLANDS.

*Bi-monthly.*

*The Philippine Journal of Science.* Manila: Bureau of Printing.

### AUSTRALASIA.

### AUSTRALIA.

*Weekly.*

*The Australian Medical Journal.* Melbourne: Charles J. Steel.

### NEW ZEALAND.

*Quarterly.*

*New Zealand Medical Journal.* Wellington: B.M.A. (Wellington Branch).

### EUROPE

### AUSTRIA.

*Weekly.*

*Wiener klinische Wochenschrift.* Vienna: Wilhelm Braumüller.

### BELGIUM.

*Monthly.*

*Bulletin de l'Académie royale de Médecine de Belgique* Brussels: F. Hayez.

### BRITISH ISLES.

*Weekly.*

*British Medical Journal.* London: 429 Strand.

*Medical Press and Circular.* London: A. A. Tindal.

*The Clinical Journal.* London: The Medical Publishing Company, Limited.

*The Hospital.* London: The Scientific Press (Limited).

*The Lancet.* London: 423 Strand.

*The Sanitary Record.* London: The Sanitary Publishing Co., Ltd.

*Fortnightly.*

*The Journal of Tropical Medicine.* London: John Bale, Sons & Danielsson, Ltd.

*Monthly.*

*Edinburgh Medical Journal.* Edinburgh: Wm. Green & Sons.

*Public Health.* London: The Society of Medical Officers of Health.

*The Birmingham Medical Review.* Birmingham: Percival Jones, Ltd.

*The British Journal of Dermatology.* London: H. K. Lewis.

*The Glasgow Medical Journal.* Glasgow: Alex. Macdougall.

*The Journal of Laryngology, Rhinology, and Otology.* London: Adlard & Son.

*The Journal of Vaccine Therapy.* London: H. K. Lewis.

*The Medical Review.* London: 66 Finsbury Pavement.

*The Medical Chronicle.* Manchester: Sherratt & Hughes.

*The Polyclinic.* London: John Bale, Sons & Danielsson, Ltd.

*The Practitioner.* London: Howard House, Strand.

*The Prescriber.* Edinburgh: 137 George Street.

*The Proceedings of the Royal Society of Medicine.* London: Longmans, Green & Co

*Quarterly.*

*Journal of Mental Science.* London: J. & A. Churchill.

*The British Journal of Tuberculosis.* London: Baillière, Tindall & Cox.

*The Caledonian Medical Journal.* Glasgow: Alex. Macdougall.

*The Northumberland and Durham Medical Journal.* Newcastle-upon-Tyne: T. M. Grierson.

*The West London Medical Journal.* London: Adlard & Son.

*Half-yearly.*

*The Liverpool Medico-Chirurgical Journal.* Liverpool: Medical Institution.

*Yearly.*

*Guy's Hospital Reports.* London: J. & A. Churchill.

*King's College Hospital Reports.* London: Adlard & Son.

*Ophthalmological Transactions.* London: J. & A. Churchill.

*St. Bartholomew's Hospital Reports.* London: Smith, Elder & Co.

*St. Thomas's Hospital Reports.* London: J. & A. Churchill.

*The Medical Annual.* Bristol: John Wright & Co.

*The Medical Society's Transactions.* London: Harrison & Sons.

*The Transactions of the Edinburgh Obstetrical Society.* Edinburgh: Oliver and Boyd.

*The Westminster Hospital Reports.* London: Henry J. Glaisher.

*Transactions of the Edinburgh Medico-Chirurgical Society.* Edinburgh: James Thin.

*Transactions of the Royal Academy of Medicine in Ireland.* Dublin: John Falconer.

*Year-Book of Scientific and Learned Societies.* London: C. Griffin & Co., Ltd.

### DENMARK.

*Weekly.*

*Hospitalstidende.* Copenhagen: Jacob Lund.

### FRANCE.

*Three times a week.*

*Gazette des Hôpitaux.* Paris: 49 Rue St.-André-des-Arts.

*Weekly.*

*Bulletin de l'Académie de Médecine.* Paris: Masson et Cie

*Gazette des Hôpitaux de Toulouse.* Toulouse: 13 Rue Ste-Ursule.

FRANCE—*Weekly* (continued).

*Gazette hebdomadaire des Sciences médicales de Bordeaux.* Bordeaux: 8 Rue Paul-Bert.

*L'Écho médical du Nord.* Lille: 128 Boulevard de la Liberté.

*Le Progrès médical.* Paris: 41 Rue des Ecoles.

*Revue hebdomadaire de Laryngologie, d'Otologie et de Rhinologie.* Bordeaux: G. Gounouilhou.

*Fortnightly.*

*Gazette des Praticiens.* Lille: Rue des Guinguettes, 18.

*Twice a month.*

*Gazette de Gynécologie.* Paris: 8bis Rue de Châteaudun.

*Revue de Thérapeutique médico-chirurgicale.* Paris: Félix Alcan.

*Monthly.*

*Journal d'Hygiène.* Paris: 79 Avenue de Wagram.

*Revue générale d'Ophtalmologie.* Paris: Masson et Cie.

GERMANY.

*Weekly*

*Zentralblatt für Chirurgie.* Leipzig: J. A. Barth.

*Zentralblatt für Gynäkologie.* Leipzig: J. A. Barth.

*Zentralblatt für innere Medicin.* Leipzig: J. A. Barth.

*Münchener medicinische Wochenschrift.* Munich: J. F. Lehmann.

GREECE.

*Monthly.*

*Ιατρική πρόοδος.* Syra: Renieri Brindesi.

*La Grèce médicale.* Syra: Renieri Brindesi.

*Three times a year.*

*Δελτίον τῆς ἐν Ἀθήναις Ἰατρικῆς Ἑταιρείας.* Athens: 85 rue de l' Université.

HOLLAND.

*Weekly.*

*Nederlandsch Tijdschrift voor Geneeskunde.* Amsterdam: F. van Rossen.

ITALY.

*Twice a month.*

*Giornale Internazionale delle Scienze Mediche.* Naples: Enrico Detken.

*Bi-monthly.*

*Archivio di Ortopedia.* Milan: Via San Calimero, 31.

NORWAY.

*Monthly.*

*Norsk Magazin for Lægevidenskaben.* Christiania: Steen'ske Bogtrykkeri.

RUSSIA.

*Weekly*

*Medycyna.* Warsaw: Niecala, 7.

*Twice a month.*

*St. Petersburger medicinische Zeitschrift.* St. Petersburg: K. L. Ricker.

*Monthly*

*Finska Läkaresällskapets Handlingar.* Helsingfors: Helsingfors Centraltryckeri.

SPAIN.

*Weekly*

*El Siglo Médico.* Madrid: Magdalena, 36.

SWEDEN.

*Bi-monthly.*

*Nordiskt Medicinskt Arkiv.* Stockholm: P. A. Norstedt & Söner.

TURKEY.

*Monthly.*

*Gazette médicale d'Orient.* Constantinople: "Levant Herald."

---

Books for Review and Exchange Journals should be sent to the Assistant-Editor, *Bristol Medico-Chirurgical Journal*, Medical Library, The University, Bristol.

### *PUBLICATIONS RECEIVED.*

---

From EDWARD ARNOLD, London :

*Glycosuria and Allied Conditions.* By P. J. CAMMIDGE, M.D. 1913. Price, 16s. net.

*Tabular Diagnosis.* By RALPH WINNINGTON LEFTWICH, M.D. 1913. Price, 7s. 6d. net.

*Diabetes: its Pathological Physiology.* By JOHN J. R. MACLEOD, M.B., Ch.B., D.P.H. 1913. Price, 10s. 6d. net.

From CASSELL AND COMPANY, LTD., London :

*Diseases of Women.* By GEORGE ERNEST HERMAN, M.B., F.R.C.S. Enlarged edition, revised by the Author, assisted by R. DRUMMOND MAXWELL. 1913. Price, 25s.

From J. & A. CHURCHILL, London :

*Vicious Circles in Disease.* By JAMIESON B. HURRY, M.A., M.D. Second and Enlarged Edition. 1913. Price, 7s. 6d. net.

From HENRY FROWDE & HODDER & STOUGHTON, London :

*A Manual of Elementary Zoology.* By L. A. BORRADAILE, M.A. 1912. Price, 10s. 6d. net.

*The Medical Diseases of Children.* By T. R. C. WHIPHAM, M.A., M.D. [1912.] Price, 10s. 6d. net.

*Diseases of the Eyes.* By C. DEVEREUX MARSHALL, F.R.C.S. [1912.] Price, 10s. 6d. net.

*Treatment after Operation.* By WILLIAM TURNER, M.S., F.R.C.S., and E. ROCK CARLING, B.S., F.R.C.S. [1912.] Price, 10s. 6d. net.

*Minor Surgery.* By LEONARD A. BIDWELL, F.R.C.S. Second Edition. [1912.] Price, 10s. 6d. net.

*The Diseases of the Skin.* By WILLMOTT EVANS, M.D., B.S. [1912.] Price, 10s. 6d. net.

From HIRSCHFELD BROTHERS, LIMITED, London :

*The Illness and Death of Napoleon Bonaparte.* By ARNOLD CHAPLIN, M.D. 1913. Price, 2s. 6d.

From J. F. LEHMANN, Munich :

*Beiträge zur Ösophagus-Chirurgie.* Von Dr. ALWIN ACH. 1913. M. 4.

From H. K. LEWIS, London :

*Flatulence and Shock.* By F. G. CROOKSHANK, M.D. 1912. Price, 2s. net.

*Vaccine Therapy.* By R. W. ALLEN, M.D., B.S. Fourth Edition. 1912. Price, 9s. net.

*The Bacterial Diseases of Respiration, and Vaccines in their Treatment.* By R. W. ALLEN, M.D., B.S. 1913. Price, 6s. net.

*The Bradshaw Lecture on the Biology of Tumours.* By C. MANSELL MOULLIN, M.A., M.D., F.R.C.S. 1913. Price, 2s. net.

From E. & S. LIVINGSTONE, Edinburgh :

*Modern Wound Treatment, and the Conduct of an Operation.* By Sir GEORGE T. BEATSON, K.C.B., M.D. 1913. Price, 2s. net.

From STANLEY PAUL & Co., London :

*The Insanity of Genius.* By J. F. NISBET. Sixth Edition. With a Preface by BERNARD HOLLANDER, M.D. [1912.] Price, 5s. net.

From *The Prescriber* Offices, Edinburgh :

*The Prescriber.* Volume VI. 1912.

From GEORGE PULMAN & SONS, LIMITED, London :

*Sclero-Corneal Trephining in Glaucoma.* By ROBERT HENRY ELLIOT, M.D., B.S., F.R.C.S. [N.D.] Price, 7s. 6d. net.

*Eyestrain in Everyday Practice.* By SYDNEY STEPHENSON, M.B., C.M. 1913. Price, 3s. 6d. net.

From THE SUPERINTENDENT, GOVERNMENT PRINTING, Calcutta :

*Sleeping Sickness: a Summary of the Work done by the Sleeping Sickness Commission, 1908–10.* By Captain F. P. MACKIE, I.M.S. 1912. Price, 1s. 6d.

From JOHN WRIGHT & SONS LTD., Bristol :

*Notes on the Treatment of Tuberculosis.* By JOHN LAIRD, L.R.C.P. and S.I. 1912. Price, 2s. 6d.

